# Supplementary material for: On the Potential Role of the (Pseudo-) Jahn–Teller Effect in the Membrane Transport Processes: Enniatin B and Beauvericin
Source: Molecules. 2023 Aug 27;28(17):6264. doi: 10.3390/molecules28176264 (PMC10488337; doi:10.3390/molecules28176264)
Supplement: Supplementary file 1 [file molecules-28-06264-s001.zip › molecules-2577699-supplementary.pdf]

On the potential role of (Pseudo-) Jahn-Teller effect in membrane transport processes: enniatin B and beauvericin

Dagmar Štellerová, Vladimír Lukeš, Martin Breza*

Institute of Physical Chemistry and Chemical Physics, Slovak University of Technology in Bratislava, Radlinského 9, SK-812 37 Bratislava, Slovakia

Table S1. Lowest excited electronic states, corresponding excitation energies (E_{exc}) and oscillator strengths (f) in atomic units for the optimized structures of enniatin B in various charge states (q) (PJT active electron states in bold).

	C_3 symmetry			C_1 symmetry		
**q**	**Excited el. state**	**E_{exc} [eV]**	**f**	**Excited el. state**	**E_{exc} [eV]**	**f**
0	1^1A	5.368	0.008	-	-	-
	2^1A	5.378	0.006	-	-	-
	3^1A	5.396	0.007	-	-	-
	**1^1E**	**5.617**	**0.005**	-	-	-
	4^1A	5.640	0.005	-	-	-
	**2^1E**	**6.755**	**0.100**	-	-	-
	5^1A	6.769	0.095	-	-	-
	**3^1E**	**7.040**	**0.068**	-	-	-
+1	-	-	-	1^2A	1.074	0.002
	-	-	-	2^2A	1.164	0.003
	-	-	-	3^2A	1.375	0.002
	-	-	-	4^2A	1.630	0.000
	-	-	-	5^2A	1.713	0.002
	-	-	-	6^2A	2.322	0.072
	-	-	-	7^2A	2.526	0.005
	-	-	-	8^2A	2.638	0.004
	-	-	-	9^2A	3.156	0.002
	-	-	-	10^2A	3.252	0.000
–1	**1^2E**	**0.681**	**0.258**	1^2A	2.305	0.002
	**2^2E**	**0.971**	**0.007**	2^2A	2.429	0.001
	1^2A	1.035	0.005	3^2A	2.616	0.001
	2^2A	1.625	0.001	4^2A	2.664	0.006
	**3^2E**	**1.990**	**0.001**	5^2A	2.822	0.004
	**4^2E**	**2.432**	**0.002**	6^2A	3.427	0.002
	-	-	-	7^2A	3.780	0.004
	-	-	-	8^2A	3.874	0.001
	-	-	-	9^2A	4.005	0.007
	-	-	-	10^2A	4.188	0.002

Table S2. Lowest excited electronic states, corresponding excitation energies (E_{exc}) and oscillator strengths (*f*) in atomic units for the optimized structures of beauvericin in various charge states (*q*) relevant electron states (PJT active electron states in bold).

	C3 symmetry			C1 symmetry		
q	**Excited el. state**	**E_{exc} [eV]**	**f**	**Excited el. state**	**E_{exc} [eV]**	**f**
0	1^1A	5.363	0.004	-	-	-
	2^1A	5.398	0.002	-	-	-
	3^1A	5.424	0.003	-	-	-
	4^1A	5.553	0.000	-	-	-
	**1^1E**	**5.576**	**0.004**	-	-	-
	5^1A	5.583	0.002	-	-	-
	6^1A	5.645	0.001	-	-	-
	7^1A	5.689	0.002	-	-	-
	8^1A	5.747	0.003	-	-	-
+1	-	-	-	1^2A	0.880	0.004
	-	-	-	2^2A	1.050	0.001
	-	-	-	3^2A	1.089	0.000
	-	-	-	4^2A	1.126	0.001
	-	-	-	5^2A	1.210	0.000
	-	-	-	6^2A	1.259	0.000
	-	-	-	7^2A	1.269	0.001
	-	-	-	8^2A	1.326	0.000
	-	-	-	9^2A	1.367	0.000
	-	-	-	10^2A	1.589	0.006
–1	-	-	-	1^2A	0.058	0.006
	-	-	-	2^2A	0.086	0.012
	-	-	-	3^2A	0.285	0.001
	-	-	-	4^2A	0.323	0.001
	-	-	-	5^2A	0.348	0.000
	-	-	-	6^2A	0.796	0.022
	-	-	-	7^2A	0.865	0.032
	-	-	-	8^2A	0.870	0.017
	-	-	-	9^2A	1.065	0.003
	-	-	-	10^2A	1.144	0.001

Gibbs free energy in Gaussian [1]

The Gibbs free energy evaluation is of physical meaning only for structures corresponding to extremal points of their potential energy surface. It is usually calculated within an ideal gas and harmonic potential approximations within the ground electron state of the system under study.

The sum of the electronic energy (including internuclear repulsion) E_{el} and zero-point energy ZPE is denoted as E_0

$$E_0 = E_{el} + ZPE \quad \text{(S1)}$$

The internal thermal energy E is the sum

$$E = E_0 + E_t + E_r + E_v \quad \text{(S2)}$$

where E_t, E_r, and E_v are translational, rotational, and vibrational energies, respectively (see below). The enthalpy H in an ideal gas approximation is

$$H = E + k_B T \quad \text{(S3)}$$

where k_B is the Boltzmann constant and T is the absolute temperature. The Gibbs free energy G is defined as

$$\mathrm{G} = H - TS_{tot} \quad \text{(S4)}$$

where the total enthalpy S_{tot}

$$S_{tot} = S_t + S_r + S_v + S_{el} \quad \text{(S5)}$$

consists of translational S_t, rotational S_r, vibrational Sv, and electronic S_{el} contributions.

i) Contributions from translations.

The translational partition function q_t is

$$q_t = \left(\frac{2\pi m k_B T}{h^2}\right)^{3/2} \frac{k_B T}{p} \quad \text{(S6)}$$

where m is the molecular mass, h is the Planck constant, and p is the pressure. The translational entropy S_t contribution is

$$S_t = k_B\,(\ln q_t + 5/2) \quad \text{(S7)}$$

The translational energy contribution E_t is

$$E_t = \frac{3}{2} k_B T \quad \text{(S8)}$$

ii) Contributions from electronic motions

If the excited states are not accessible and the energy of the ground state is zero, the electronic partition function q_{el} is simplified to

$$q_{el} = \omega_0 \tag{S9}$$

which is simply the electronic spin multiplicity of the molecule. The electronic entropy contribution S_{el} is

$$S_{el} = k_B \ln q_{el} \tag{S10}$$

There is no temperature-dependent electronic contribution to thermal energy.

iii) Contributions from rotational motion

For a linear molecule, the rotational partition function q_r is

$$q_r = \frac{1}{\sigma_r} \frac{T}{\theta_r} \tag{S11}$$

where σ_r is the rotational symmetry number and the rotational constant

$$\theta_r = \frac{h^2}{8\pi^2 I k_B} \tag{S12}$$

where I is the moment of inertia. The rotational contribution E_r to the thermal energy of linear molecules is

$$E_r = k_B T \tag{S13}$$

For a nonlinear polyatomic molecule, the rotational partition function q_r is

$$q_r = \frac{\pi^{1/2}}{\sigma_r} \left(\frac{T^{3/2}}{(\theta_{r,x}\theta_{r,y}\theta_{r,z})^{1/2}} \right) \tag{S14}$$

where $\theta_{r,x}$, $\theta_{r,y}$, and $\theta_{r,z}$ are rotational constants related to the x, y and z axes, respectively. The corresponding rotational entropy contribution S_r is

$$S_r = k_B (\ln r + 3/2) \tag{S15}$$

and the rotational contribution to the thermal energy E_r is

$$E_r = \frac{3}{2} k_B T \tag{S16}$$

iv) Contributions from vibrational motion

Each of K vibrational modes has a characteristic vibrational temperature

$$\vartheta_{v,K} = \frac{h\nu_K}{k_B} \tag{S17}$$

where ν_K is the corresponding frequency. If the first vibrational energy is set to zero, then the overall vibrational partition function q_v is

$$q_v = \prod_K \frac{1}{1 - e^{\vartheta_{v,K}/T}} \quad \text{(S18)}$$

The vibrational entropy contribution S_v is

$$S_v = k_B \sum_K \left(\frac{\vartheta_{v,K}/T}{e^{\vartheta_{v,K}/T}} - 1 - \ln\left(1 - e^{\frac{\vartheta_{v,K}}{T}}\right) \right) \quad \text{(S19)}$$

The vibrational contribution to the thermal energy E_v is

$$E_v = k_B \sum_K \vartheta_{v,K} \left(\frac{1}{2} + \frac{1}{e^{\vartheta_{v,K}/T} - 1} \right) \quad \text{(S20)}$$

References:

1. Ochterski, JW Thermochemistry in Gaussian; Gaussian, Inc., 2020. Available online: https://gaussian.com/thermo/ (accessed on 18 August 2023).
